# Daily unhealthy food purchase and associated factors among adolescents in urban Dire Dawa, eastern Ethiopia: A community-based cross-sectional study

**DOI:** 10.1371/journal.pone.0359580

**Published:** 2026-10-06

**Authors:** Bira Belay, Alamiya Abdo

**Affiliations:** 1 School of Public Health, College of Health and Medical Sciences, Haramaya University, Harar, Ethiopia; 2 Department of Public Health, Harar Health Science College, Harar, Ethiopia; University of Tabuk, SAUDI ARABIA

## Abstract

**Background:**

Unhealthy food consumption is a growing concern among adolescents in low- and middle-income countries. Evidence on unhealthy food purchase in eastern Ethiopia remains limited. This cross-sectional study assessed the prevalence of daily unhealthy food purchase and associated factors among adolescents in urban Dire Dawa, eastern Ethiopia.

**Methods:**

A community-based cross-sectional study was conducted from October to November 2024 among 475 adolescents (aged 10–19 years) in urban kebeles of Dire Dawa, using multistage sampling. Daily purchase of unhealthy foods was assessed using a 5-point frequency scale and dichotomized as daily versus less than daily. Bivariable and multivariable logistic regression identified associated factors. Ordinal logistic regression was performed as sensitivity analysis.

**Results:**

Daily purchase of unhealthy foods was reported by 70 adolescents (14.7%; 95% CI: 11.6 to 18.2%). The most commonly purchased items were cakes (18.1%), traditional sweets (17.1%), and burgers (17.1%). In multivariable analysis, high availability of unhealthy foods (AOR = 3.42, 95% CI: 1.87 to 6.25), vendor influence (AOR = 2.56, 95% CI: 1.48 to 4.42), peer influence (AOR = 2.13, 95% CI: 1.24 to 3.66), poor nutrition knowledge (AOR = 2.01, 95% CI: 1.18 to 3.42), and high family income (AOR = 1.89, 95% CI: 1.08 to 3.31) were significantly associated with daily purchase. Ordinal regression confirmed similar direction, with peer influence remaining significant. Limitations include the cross-sectional design, omission of sugary beverages, and urban-only sample.

**Conclusions:**

Approximately one in seven adolescents in urban Dire Dawa reported daily purchase of unhealthy foods. Food environment factors, particularly availability and vendor influence, were the strongest predictors. Interventions should prioritize regulating unhealthy food availability near schools, strengthening nutrition education, and leveraging peer networks. Policies should address imported ultra-processed foods and sugary beverages.

## Introduction

Unhealthy food consumption among adolescents is a growing public health concern globally, particularly in low- and middle-income countries (LMICs) where nutrition transition is accelerating [1]. Unhealthy foods, typically high in added sugars, unhealthy fats, and sodium but low in essential nutrients, contribute to overweight, obesity, and non-communicable diseases [2]. Adolescence (10–19 years) represents a critical developmental window during which dietary habits are formed and often track into adulthood, making it a priority period for nutrition interventions [2,3].

In Ethiopia, the nutrition transition has brought significant changes to food environments. Traditional diets based on whole grains, legumes, and vegetables are increasingly displaced by ultra-processed foods, sugary products, and fast foods, particularly in urban areas [4]. Dire Dawa City Administration, located in eastern Ethiopia, is uniquely affected by this transition. As a major trade hub bordering Somalia and Djibouti, the city has exceptionally high availability of imported processed and ultra-processed foods compared to other Ethiopian cities [5]. Adolescents in this setting are exposed to both traditional Ethiopian foods and Western-style processed products, creating a distinct food environment that has not been studied previously.

Adolescents are particularly vulnerable to unhealthy food purchase and consumption due to multiple interacting factors. At the individual level, nutrition knowledge, taste preferences, age, and sex influence food choices [6]. At the interpersonal level, peer influence and parental modeling shape dietary behaviors [7]. At the environmental level, food availability, vendor proximity to schools, and targeted marketing create an obesogenic environment that promotes unhealthy choices [4,8]. At the socioeconomic level, family income and maternal education affect both purchasing power and nutritional awareness [9].

Home-prepared food represents an important source of adolescent dietary intake that this study does not measure. In Ethiopian households, particularly in traditional settings, home-prepared foods constitute a substantial proportion of adolescent dietary intake. The relationship between food purchase and overall dietary quality may therefore be moderated by the home food environment.

Despite growing concern, evidence on unhealthy food purchase patterns and their determinants among adolescents in eastern Ethiopia remains extremely limited. Most Ethiopian studies have focused on Addis Ababa or school-based populations in southern Ethiopia, with no studies from the eastern region's unique trade-hub setting [4,10]. Understanding these factors is essential for designing context-specific, effective interventions. This study aimed to: (1) assess the prevalence of daily unhealthy food purchase; (2) identify the most commonly purchased unhealthy foods; and (3) examine factors associated with daily unhealthy food purchase among adolescents in urban Dire Dawa, eastern Ethiopia.

## Methods

### Study design and setting

A community-based cross-sectional study was conducted from October 1 to November 30, 2024, in Dire Dawa City Administration, eastern Ethiopia. Dire Dawa is located 515 kilometers east of Addis Ababa, bordering Somalia and Djibouti. The city has an estimated population of 551,000 across nine urban kebeles (the smallest administrative units) [11]. As a major trade hub along international routes, Dire Dawa has a unique food environment characterized by high penetration of imported processed foods, numerous street vendors, and a mix of traditional and modern retail outlets. This makes it an important sentinel site for studying adolescent food purchase behaviors in the context of nutrition transition.

### Source and study population

The source population comprised all adolescents aged 10–19 years residing in the nine urban kebeles of Dire Dawa City Administration. Eligible participants were permanent residents (6 months or more of residence) who were willing to participate and provided appropriate consent or assent. Adolescents with communication disabilities (deafness, mutism, severe speech impairment) or severe chronic illnesses that would preclude interview participation were excluded.

### Sample size and sampling

Sample size was calculated using the single population proportion formula with the following assumptions: p = 50% (conservative estimate given limited prior evidence from this setting), 95% confidence interval, and 5% margin of error. This yielded 384 participants. A design effect of 1.5 was applied based on WHO recommendations for community-based nutrition surveys using multistage sampling [12]. Accounting for 10% anticipated non-response, the final sample size was 476 participants.

Multistage sampling was employed. In the first stage, four kebeles were randomly selected from the nine urban kebeles using a lottery method. In the second stage, households within each selected kebele were sampled using systematic random sampling. The sampling interval was determined by dividing the estimated number of households with adolescents by the required sample per kebele. The starting household was randomly selected. When a selected household had more than one eligible adolescent, one was randomly selected using the Kish method. If no eligible adolescent was present at the selected household, the next household was approached.

### Data collection instruments

Data were collected through face-to-face interviews using a structured questionnaire adapted from validated studies [13,14]. The questionnaire was developed in English, translated into three local languages (Amharic, Afaan Oromo, and Af-Somali) by certified translators, and back-translated to English to ensure conceptual equivalence, following the guidelines of Beaton et al. (2000) [15].

### Outcome variable

The primary outcome variable was daily purchase of unhealthy foods, assessed using the question: “In the past 7 days, how often did you purchase unhealthy foods (such as sweets, chips, cakes, burgers, pizza, traditional sweets) for your own consumption?” Response categories were: never, 1–2 times per week, 3–4 times per week, 5–6 times per week, and daily.

For the primary analysis, the outcome was dichotomized as daily purchase (coded as 1) versus less than daily purchase (coded as 0). The daily threshold was selected because it represents habitual, regular exposure to unhealthy foods of greater public health concern than occasional consumption. Previous studies have identified daily consumption of ultra-processed foods as a risk factor for adolescent overweight and metabolic syndrome [1,2]. As a sensitivity analysis, ordinal logistic regression was also conducted using the full 5-point frequency scale.

Unhealthy foods were defined as energy-dense, nutrient-poor foods high in added sugars, unhealthy fats, and/or sodium. The specific food items assessed were chocolates, chips (crisps), traditional sweets (beklewa), cakes, burgers, and pizza. We acknowledge that sugary beverages were not included in the original questionnaire. This represents a fundamental validity threat discussed in the Limitations section.

### Independent variables

Independent variables were grouped into four categories:

**Sociodemographic factors:** Age (categorized as 10–13, 14–16, and 17–19 years), sex (male or female), family income (low: 10,000 ETB or less per month; middle: 10,001–20,000 ETB per month; high: 20,001 ETB or more per month), and maternal education (illiterate, can read and write, primary, secondary, college and above).
**Psychosocial factors:** Peer influence (assessed by the question: “Do your friends encourage or influence you to eat unhealthy foods?” with yes or no response) and parental influence (assessed by the question: “Do your parents or guardians influence what you eat?” with yes or no response). Single-item questions were selected because the study was community-based with time constraints, and there are currently no validated multi-item scales for measuring peer and parental influence on food purchase culturally adapted for Ethiopian adolescents. We acknowledge that single-item measures may not fully capture the multidimensional nature of these constructs.
**Food environment factors:** Unhealthy food availability (assessed by asking adolescents about the number of locations selling unhealthy foods within walking distance of their home or school; categorized as low [0–1 locations], moderate [2–3 locations], or high [4 or more locations]) and vendor influence (assessed by the question: “Do food vendors or advertisements influence you to buy unhealthy foods?” with yes or no response).
**Nutrition-related factors:** Nutrition knowledge (assessed using a 9-item scale covering knowledge of healthy eating, food groups, and diet-disease relationships; total score range 0–9; categorized as good [score 5 or more] or poor [score less than 5], consistent with previous Ethiopian studies [4,10]) and food security status (assessed using the validated Household Food Insecurity Access Scale [HFIAS] and categorized as secure or insecure) [16].

### Data quality control

The questionnaire underwent content validation by a panel of three nutrition experts at Haramaya University. A pretest was conducted on 5% (n = 24) of the sample in a kebele not selected for the main study. The pretest assessed clarity, comprehensibility, cultural appropriateness, and time to complete. Internal consistency for the nutrition knowledge scale was acceptable (Cronbach's alpha = 0.78). Test-retest reliability assessed over a 2-week interval among 30 adolescents yielded kappa values ranging from 0.65 to 0.82 for key categorical variables.

Four data collectors (all holding bachelor's degrees in public health or nutrition) and two supervisors (MPH holders) were trained for two days on the study objectives, questionnaire administration, ethical procedures, and data quality protocols. Daily supervision, on-site review of completed questionnaires for completeness, and double data entry with validation checks were performed.

### Statistical analysis

Data were analyzed using R software version 4.3.2 [17]. Descriptive statistics were calculated for all variables. Categorical variables were summarized as frequencies and percentages; continuous variables were summarized as means with standard deviations.

Bivariable logistic regression was performed to identify candidate variables associated with daily unhealthy food purchase. Variables with a p-value less than 0.25 in bivariable analysis were entered into the multivariable logistic regression model. This liberal threshold was selected based on the recommendation of Hosmer and Lemeshow (2000) to avoid excluding potentially important confounders at the screening stage [18].

Multicollinearity among independent variables was assessed using variance inflation factor (VIF); all VIF values were below 2.0, indicating no significant collinearity. Model fit was assessed using the Hosmer-Lemeshow goodness-of-fit test. Adjusted odds ratios (AOR) with 95% confidence intervals (CI) were reported. Statistical significance was declared at p less than 0.05.

We acknowledge that we did not apply correction for multiple comparisons. This decision was made because the study was exploratory in nature, conducted in a setting with no prior evidence on adolescent unhealthy food purchase. Applying strict corrections such as Bonferroni would increase the risk of Type II error. However, we recognize that with five independent variables and a significance threshold of p less than 0.05, the probability of at least one false positive is approximately 22.6%. Our findings should therefore be interpreted as hypothesis-generating and require replication in independent samples.

As a sensitivity analysis, ordinal logistic regression (proportional odds model) was conducted using the full 5-point frequency scale as the outcome. The proportional odds assumption was tested using the Brant test. Results are presented in S1 Table.

### Ethical considerations

This study was conducted in accordance with the Declaration of Helsinki. Ethical approval was obtained from the Institutional Health Research Ethics Review Committee (IHRERC) of Haramaya University (reference number: IHRERC/259/2024). Written informed consent was obtained from all participants aged 18 years and older. For participants aged 10–17 years, written informed assent was obtained from the adolescent, and written informed consent was obtained from their parent or legal guardian. All participants were informed about the study purpose, voluntary nature of participation, confidentiality protections, and the right to withdraw at any time without consequence.

## Results

### Participant characteristics

Of 476 eligible participants, 475 were interviewed, yielding a response rate of 99.6%. The mean age was 14.4 years (SD = 2.9). Over half (51.6%) were male, and 41.7% were in the younger adolescent age group (10–13 years). The majority of families (64.8%) had a middle-level monthly income (10,001–20,000 ETB). Good nutrition knowledge was observed in 54.5% of participants. High availability of unhealthy foods (4 or more locations within walking distance) was reported by 33.9% of adolescents. Food insecurity affected 29.5% of households (Table 1).

**Table 1:** Participant characteristics among adolescents in urban Dire Dawa, eastern Ethiopia.

Characteristic	Category	Frequency	Percentage
Age (years)	10–13	198	41.7
	14–16	136	28.6
	17–19	141	29.7
Sex	Male	245	51.6
	Female	230	48.4
Family income (ETB)	Low (10,000 or less)	152	32
	Middle (10,001 to 20,000)	308	64.8
	High (20,001 or more)	15	3.2
Maternal education	Illiterate	168	35.4
	Can read and write	65	13.7
	Primary	156	32.8
	Secondary	67	14.1
	College and above	19	4
Nutrition knowledge	Poor (score less than 5)	216	45.5
	Good (score 5 or more)	259	54.5
Peer influence	No	212	44.6
	Yes	263	55.4
Parental influence	No	170	35.8
	Yes	305	64.2
Unhealthy food availability	Low (0–1 locations)	124	26.1
	Moderate (2–3 locations)	190	40
	High (4 or more locations)	161	33.9
Vendor influence	No	244	51.4
	Yes	231	48.6
Food security	Insecure	140	29.5
	Secure	335	70.5

### Prevalence of daily unhealthy food purchase

Daily purchase of unhealthy foods was reported by 70 adolescents, yielding a prevalence of 14.7% (95% CI: 11.6 to 18.2%). The full distribution of purchase frequency was: never, 113 (23.8%); 1–2 times per week, 144 (30.3%); 3–4 times per week, 86 (18.1%); 5–6 times per week, 70 (14.7%); and daily, 62 (13.1%).

### Types of unhealthy foods purchased

Among all adolescents, the most commonly purchased unhealthy foods (ever in the past week) were cakes (n = 86, 18.1%), traditional sweets/beklewa (n = 81, 17.1%), burgers (n = 81, 17.1%), chocolates (n = 79, 16.6%), pizza (n = 77, 16.2%), and chips/crisps (n = 71, 14.9%).

### Factors associated with daily unhealthy food purchase

In bivariable analysis, variables meeting the p less than 0.25 criterion for inclusion in the multivariable model were age, sex, family income, nutrition knowledge, peer influence, parental influence, unhealthy food availability, vendor influence, and food security status.

In multivariable analysis, five factors remained significantly associated with daily unhealthy food purchase after adjusting for all other variables (Table 2).

**Table 2:** Factors associated with daily unhealthy food purchase among adolescents in urban Dire Dawa, eastern Ethiopia.

Variable	Category	AOR	95% CI	p-value
**Unhealthy food availability**	Low (0–1)	1.00	Reference	—
	Moderate (2–3)	1.56	0.82–2.97	0.176
	High (4 or more)	3.42	1.87–6.25	<0.001
**Vendor influence**	No	1.00	Reference	—
	Yes	2.56	1.48–4.42	0.001
**Peer influence**	No	1.00	Reference	—
	Yes	2.13	1.24–3.66	0.006
**Nutrition knowledge**	Good (score ≥5)	1.00	Reference	—
	Poor (score <5)	2.01	1.18–3.42	0.010
**Family income (ETB)**	Low (≤10,000)	1.00	Reference	—
	Middle (10,001–20,000)	1.48	0.82–2.68	0.196
	High (≥20,001)	1.89	1.08–3.31	0.025

**AOR**, Adjusted Odds Ratio; **CI**, Confidence Interval; **ETB**, Ethiopian Birr.

Model fit: Hosmer-Lemeshow χ² = 7.34, df = 8, p = 0.501. No multicollinearity (all VIF < 2.0).

Adolescents who reported high availability of unhealthy foods had 3.42 times higher odds of daily purchase compared to those with low availability (AOR = 3.42, 95% CI: 1.87 to 6.25, p less than 0.001). Moderate availability was not significantly associated (AOR = 1.56, 95% CI: 0.82 to 2.97, p = 0.176).

Adolescents who reported being influenced by food vendors had 2.56 times higher odds of daily unhealthy food purchase compared to those not influenced (AOR = 2.56, 95% CI: 1.48 to 4.42, p = 0.001).

Peer influence was associated with 2.13 times higher odds of daily unhealthy food purchase (AOR = 2.13, 95% CI: 1.24 to 3.66, p = 0.006).

Adolescents with poor nutrition knowledge had 2.01 times higher odds of daily unhealthy food purchase compared to those with good knowledge (AOR = 2.01, 95% CI: 1.18 to 3.42, p = 0.010).

Adolescents from high-income families had 1.89 times higher odds of daily unhealthy food purchase compared to those from low-income families (AOR = 1.89, 95% CI: 1.08 to 3.31, p = 0.025). Middle income was not significantly associated (AOR = 1.48, 95% CI: 0.82 to 2.68, p = 0.196).

The Hosmer-Lemeshow goodness-of-fit test indicated adequate model fit (chi-square = 7.34, df = 8, p = 0.501). No multicollinearity was detected (all VIF less than 2.0).

### Sensitivity analysis

Ordinal logistic regression using the full 5-point purchase frequency scale was conducted as a sensitivity analysis. The proportional odds assumption was met (Brant test omnibus p = 0.76). Peer influence remained significantly associated with more frequent unhealthy food purchase (proportional OR = 1.40, 95% CI: 1.01 to 1.95, p = 0.043). High availability of unhealthy foods showed a consistent direction of association (proportional OR = 1.41, 95% CI: 0.92 to 2.18) but was attenuated compared to the binary analysis. Vendor influence, nutrition knowledge, and family income showed attenuated associations in the ordinal model compared to the binary analysis.

The attenuation of effects in the ordinal model compared to the binary model suggests that these factors may be particularly important for daily habitual purchase rather than occasional purchase. The binary analysis focusing on daily versus less than daily purchase may capture a threshold effect that is diluted when averaging across all frequency categories in the ordinal model (S1 Table).

## Discussion

This study provides the first evidence on daily unhealthy food purchase and its determinants among adolescents in urban Dire Dawa, a major trade hub in eastern Ethiopia. We found that 14.7% of adolescents reported purchasing unhealthy foods daily. While this prevalence is lower than that reported from Addis Ababa, where studies have found up to 25–30% of adolescents regularly consuming ultra-processed foods [4], it is higher than estimates from rural Ethiopian settings, where such behaviors are rare [10]. The intermediate prevalence likely reflects urban Dire Dawa's position as a trade hub undergoing nutrition transition: more exposed than rural areas but less saturated than the capital.

The most commonly purchased unhealthy foods were cakes (18.1%), traditional sweets/beklewa (17.1%), and burgers (17.1%). The prominence of cakes and burgers reflects the penetration of Western-style fast foods into the local food environment, while the high purchase of beklewa (traditional sweets) indicates that locally produced unhealthy foods remain important. This dual burden of imported and traditional unhealthy foods is characteristic of nutrition transition settings where global and local food systems intersect [1].

Sugar-sweetened beverages (SSBs), including sodas, sweetened juices, and energy drinks, represent the most commonly consumed unhealthy items among adolescents globally. Their omission from our questionnaire is a significant limitation that likely leads to a substantial underestimate of total unhealthy dietary exposure in our study population. The prevalence estimate of 14.7% for daily unhealthy food purchase is therefore likely a substantial underestimate of total unhealthy dietary exposure when beverages are considered. Policy recommendations should specifically address SSB consumption through taxation, marketing restrictions, and school-based bans.

Our study focused exclusively on food purchase behavior and did not assess home-prepared food consumption. In Ethiopian households, particularly in traditional settings, home-prepared foods represent a substantial proportion of adolescent dietary intake. The relationship between food purchase and overall dietary quality may therefore be moderated by the home food environment. Adolescents who purchase unhealthy foods daily may still consume adequate home-prepared meals, or conversely, those who do not purchase unhealthy foods may consume unhealthy home-prepared foods. Future studies should incorporate comprehensive dietary assessment including home-prepared foods, purchased foods, and foods consumed at school.

### Food environment factors

High availability of unhealthy foods was the strongest predictor of daily purchase (AOR = 3.42), consistent with ecological models emphasizing that the food environment shapes dietary behaviors [6]. In urban Dire Dawa, the strategic location as a trade hub facilitates the influx of imported processed foods at competitive prices. Adolescents with four or more unhealthy food outlets within walking distance of their homes or schools had substantially higher odds of daily purchase. This finding supports a growing body of evidence linking food environment characteristics, particularly outlet density and proximity to schools, to adolescent dietary behaviors [4,8]. From a policy perspective, this suggests that zoning regulations limiting unhealthy food outlets near schools could be effective in this setting.

Vendor influence was the second strongest predictor (AOR = 2.56). Food vendors in urban Dire Dawa often employ targeted marketing strategies including attractive packaging, affordable pricing, and strategic placement near schools and youth gathering points. This finding aligns with studies from other LMIC settings where vendor marketing has been identified as a key driver of adolescent unhealthy food choices [4,7]. The absence of regulations on food marketing to children in Ethiopia makes adolescents particularly vulnerable to vendor influence.

### Interpersonal and individual factors

Peer influence was significantly associated with daily unhealthy food purchase (AOR = 2.13), consistent with the well-established role of peer social norms in shaping adolescent eating behaviors [6,7]. During adolescence, peer approval and conformity become powerful motivators of behavior. Friends often share food purchases, recommend vendors, and create social norms around unhealthy food consumption. This finding suggests that peer-led nutrition interventions, in which adolescents themselves become advocates for healthy eating, could be particularly effective in eastern Ethiopian schools.

Poor nutrition knowledge was associated with twice the odds of daily unhealthy food purchase (AOR = 2.01). Adolescents with limited understanding of healthy eating, food groups, and the health consequences of poor diet may be less equipped to resist the appeal of unhealthy foods. This finding reinforces the importance of school-based nutrition education, which remains underimplemented in Ethiopian schools [5,10].

### Socioeconomic factors

High family income was associated with 1.89 times higher odds of daily unhealthy food purchase. This likely reflects greater discretionary income available for non-essential food purchases. This finding is notable because it indicates that unhealthy food purchase in urban Dire Dawa is not primarily a problem of food insecurity or poverty, but rather one of food choice in the context of increasing purchasing power. Similar patterns have been observed in other rapidly urbanizing settings where economic growth has preceded nutrition literacy [1,9,19].

### Limitations

This study has several limitations. First, the cross-sectional design precludes causal inference. The observed associations should not be interpreted as causal relationships. For example, adolescents with pre-existing preferences for unhealthy foods may seek out environments with greater availability and vendor presence, rather than the environment causing the behavior. Longitudinal studies are needed to establish temporality.

Second, we assessed unhealthy food purchase rather than actual consumption. Purchase may not equate to consumption, as purchased foods may be shared or not fully consumed. Conversely, adolescents who do not purchase food may still consume unhealthy foods at home.

Third, the binary classification (daily versus less than daily) loses information compared to the full ordinal scale. While our sensitivity analysis using ordinal logistic regression confirmed the direction of association for most variables, the effects were attenuated. Only peer influence remained statistically significant in the ordinal model. This discrepancy suggests that the predictors may be specifically associated with daily habitual purchase rather than less frequent purchase.

Fourth, the omission of sugary beverages represents a fundamental validity threat to our estimate of unhealthy food purchase. Sugar-sweetened beverages are the most commonly consumed unhealthy items among adolescents globally, and their absence from our questionnaire means that our prevalence estimate is likely a substantial underestimate of total unhealthy dietary exposure.

Fifth, self-reported data may be subject to recall bias and social desirability bias. Adolescents may underreport unhealthy food purchase due to perceived social undesirability.

Sixth, our sample was drawn exclusively from urban kebeles of Dire Dawa. The findings are not generalizable to rural areas of eastern Ethiopia, where food environments differ substantially.

Seventh, residual confounding from unmeasured variables cannot be excluded. We did not measure media exposure, screen time, body image concerns, physical activity levels, or home food environment characteristics.

Eighth, we did not apply correction for multiple comparisons. With five independent variables and a significance threshold of p less than 0.05, the probability of at least one false positive is approximately 22.6%. Our findings should be interpreted as hypothesis-generating and require replication in independent samples.

Ninth, peer and parental influence were assessed using single-item questions, which may not fully capture the multidimensional nature of these constructs. Future studies should employ validated multi-item scales culturally adapted for Ethiopian adolescents.

### Implications for policy and practice

Despite these limitations, our findings have several implications. First, the strong association between food availability and daily unhealthy food purchase supports policies to regulate the density and proximity of unhealthy food outlets, particularly around schools. Zoning regulations and healthy school food zones have been effective in other settings [20].

Second, the influence of vendors underscores the need for marketing regulations. Ethiopia currently lacks comprehensive policies restricting food marketing to children, a gap that our findings suggest should be addressed urgently.

Third, school-based nutrition education programs should be strengthened and expanded. Peer-led approaches that leverage adolescents’ social networks may be particularly effective.

Fourth, given urban Dire Dawa's unique position as a trade hub, specific policies addressing the influx of imported ultra-processed foods, such as front-of-package warning labels or import taxation, should be considered.

Fifth, interventions should specifically address sugar-sweetened beverage consumption through taxation, marketing restrictions, and school-based bans.

## Conclusion

Approximately one in seven adolescents in urban Dire Dawa, eastern Ethiopia, reported purchasing unhealthy foods daily. High availability of unhealthy foods, vendor influence, peer influence, poor nutrition knowledge, and high family income were independently associated with daily purchase. Food environment factors, particularly availability and vendor influence, were the strongest predictors, highlighting the need for structural interventions alongside individual-level nutrition education. In urban Dire Dawa's unique trade-hub context, policies should address both imported and locally produced unhealthy foods, with particular attention to regulating the food environment around schools and addressing sugar-sweetened beverage consumption. Longitudinal studies incorporating comprehensive dietary assessment including beverages and home-prepared foods are needed to establish causal pathways and inform intervention design.

## Supporting information

S1 Table Ordinal logistic regression results for unhealthy food purchase frequency (N = 475). Proportional odds ratios estimated from ordinal logistic regression. The proportional odds assumption was met (Brant test omnibus p = 0.76).
(DOCX)
