# Supplementary material for: Daily unhealthy food purchase and associated factors among adolescents in urban Dire Dawa, eastern Ethiopia: A community-based cross-sectional study
Source: PLoS One. 2026 Oct 6;21(10):e0359580. doi: 10.1371/journal.pone.0359580 (PMC13641702; doi:10.1371/journal.pone.0359580)
Supplement: Supplementary file 1 [file pone.0359580.s001.docx]

**Table S1:** Ordinal Logistic Regression Results for Unhealthy Food Purchase Frequency (N = 475)]

| Variable | Proportional_OR | CI_Lower | CI_Upper | p_value |
| --- | --- | --- | --- | --- |
| Unhealthy food availability (ref: Low) |  |  |  |  |
| Moderate | 1.05 | 0.69 | 1.58 | 0.8274 |
| High | 1.41 | 0.92 | 2.18 | 0.1183 |
| Vendor influence (ref: No) |  |  |  |  |
| Yes | 0.87 | 0.63 | 1.21 | 0.4167 |
| Peer influence (ref: No) |  |  |  |  |
| Yes | 1.4 | 1.01 | 1.95 | 0.0431 |
| Nutrition knowledge (ref: Poor) |  |  |  |  |
| Good | 0.97 | 0.7 | 1.35 | 0.8613 |
| Family income (ref: Low) |  |  |  |  |
| Middle | 1.25 | 0.88 | 1.78 | 0.2177 |
| High | 0.91 | 0.35 | 2.35 | 0.8431 |
| Age (ref: 10-13) |  |  |  |  |
| 14-16 | 0.99 | 0.67 | 1.48 | 0.9683 |
| 17-19 | 1.06 | 0.72 | 1.57 | 0.7689 |
| Sex (ref: Male) |  |  |  |  |
| Female | 0.92 | 0.66 | 1.27 | 0.6138 |
| Parental influence (ref: No) |  |  |  |  |
| Yes | 0.92 | 0.65 | 1.29 | 0.618 |
| Food security (ref: Insecure) |  |  |  |  |
| Secure | 0.96 | 0.67 | 1.38 | 0.8324 |
